# Laboratory recommendations for scoring deep molecular responses following treatment for chronic myeloid leukemia

**DOI:** 10.1038/leu.2015.29

**Published:** 2015-02-27

**Authors:** N C P Cross, H E White, D Colomer, H Ehrencrona, L Foroni, E Gottardi, T Lange, T Lion, K Machova Polakova, S Dulucq, G Martinelli, E Oppliger Leibundgut, N Pallisgaard, G Barbany, T Sacha, R Talmaci, B Izzo, G Saglio, F Pane, M C Müller, A Hochhaus

**Affiliations:** 1 Faculty of Medicine, University of Southampton, Southampton, UK; 2 National Genetics Reference Laboratory (Wessex), Salisbury District Hospital, Salisbury, UK; 3 Hematopathology Unit, Hospital Clinic, IDIBAPS, Barcelona, Spain; 4 Department of Clinical Genetics, Laboratory Medicine, Lund University, Lund, Sweden; 5 Imperial Molecular Pathology Laboratory, Centre for Haematology, Imperial College London, London, UK; 6 Department of Clinical and Biological Science, University of Turin, Turin, Italy; 7 Abteilung für Hämatologie und internistische Onkologie, Universität Leipzig, Leipzig, Germany; 8 Children's Cancer Research Institute/LabDia Labordiagnostik and Medical University, Vienna, Austria; 9 Institute of Hematology and Blood Transfusion, Prague, Czech Republic; 10 Laboratoire Hematologie, CHU Bordeaux, Hematopoiese Leucemique et Cibles Therapeutiques, INSERM U1035, Université Bordeaux, Bordeaux, France; 11 Department of Experimental, Diagnostic and Specialty Medicine, University of Bologna, Bologna, Italy; 12 Molecular Diagnostics Laboratory, Department of Hematology, University Hospital Bern, Bern, Switzerland; 13 Klinisk Biokemi, Vejle Sygehus, Vejle, Denmark; 14 Department of Molecular Medicine and Surgery, Clinical Genetics, Karolinska Institutet, Stockholm, Sweden; 15 Hematology Department, Jagiellonian University, Krakow, Poland; 16 Hematology Department – Fundeni Clinical Institute, University of Medicine and Pharmacy ‘Carol Davila', Bucharest, Romania; 17 Department of Clinical Medicine and Surgery, University ‘Federico II' of Naples, Naples, Italy; 18 CEINGE – Biotecnologie Avanzate, Naples, Italy; 19 III. Medizinische Klinik, Universitätsmedizin Mannheim, Mannheim, Germany; 20 Abteilung Hämatologie/Onkologie, Klinik für Innere Medizin II, Universitätsklinikum Jena, Jena, Germany

## Abstract

Treatment of chronic myeloid leukemia (CML) with tyrosine kinase inhibitors has advanced to a stage where many patients achieve very low or undetectable levels of disease. Remarkably, some of these patients remain in sustained remission when treatment is withdrawn, suggesting that they may be at least operationally cured of their disease. Accurate definition of deep molecular responses (MRs) is therefore increasingly important for optimal patient management and comparison of independent data sets. We previously published proposals for broad standardized definitions of MR at different levels of sensitivity. Here we present detailed laboratory recommendations, developed as part of the European Treatment and Outcome Study for CML (EUTOS), to enable testing laboratories to score MR in a reproducible manner for CML patients expressing the most common *BCR-ABL1* variants.

## Introduction

Molecular monitoring provides important prognostic information for individual chronic myeloid leukemia (CML) patients undergoing therapy, and international treatment recommendations incorporate specific time-dependent molecular milestones to help determine whether a patient is responding optimally or not.^1, 2^ Molecular measurements are made by reverse transcriptase quantitative PCR (RT-qPCR) to estimate the amount of *BCR-ABL1* mRNA relative to an internal reference gene, most commonly *ABL1*, *GUSB* or *BCR*.^3, 4^ The results are expressed on an International Scale (IS) as a percentage, with 100% BCR-ABL^IS^ corresponding to the International Randomized Study of Interferon and STI571 (IRIS) study standardized baseline and 0.1% BCR-ABL^IS^ being defined as a major molecular response (MMR or MR^3^; 3 log reduction from the standardized baseline).^3^ Expression of results on the IS depends on each testing laboratory either having obtained a laboratory-specific conversion factor (CF) by sample exchange with an established reference laboratory or by using kits and reagents that have been calibrated to the World Health Organization International Genetic Reference Panel for quantitation of *BCR-ABL1* mRNA.^4, 5, 6, 7, 8, 9^

Efforts to standardize molecular monitoring to the IS focused initially on detectable residual disease and in particular whether a patient had or had not achieved particular milestones, for example, 10% BCR-ABL^IS^ or 0.1% BCR-ABL^IS^ at various time points. However, with longer follow-up, it became apparent that many patients treated with imatinib achieved deeper levels of response, with *BCR-ABL1* becoming undetectable in a minority of cases.^10^ This, along with the fact that second-generation tyrosine kinase inhibitors produce faster and deeper responses, compared with imatinib,^11, 12^ prompted the need for robust, standardized definitions of deep MR. Such definitions are particularly important in the context of studies that are enrolling patients with sustained deep responses into treatment-free protocols.^13, 14^

We previously published proposals for broad standardized definitions of MR at different levels of sensitivity (MR^4^, MR^4.5^, and so on; collectively referred to as ‘deep MR'), which were endorsed by the European LeukemiaNet in their most recent recommendations for the treatment of CML patients.^1, 15^ These broad definitions, however, and clinical studies that have been published to date do not provide the technical details and interpretation to enable laboratories to categorize patients in a standardized manner. As part of the European Treatment and Outcome Study (EUTOS), we have developed laboratory proposals, as detailed below, to enable testing laboratories to define MR in a reproducible manner. These proposals were developed by consensus over several meetings and are described in detail in this paper, along with several examples. The terminology employed is based on the recommendations of the Minimum Information for Publication of Quantitative Real-Time PCR Experiments (MIQE) guidelines^16^ and the proposal focuses on qPCR assays for the most common *BCR-ABL1* variants (e13a2 and/or e14a2; 97% of CML patients) that use an external plasmid calibrator to estimate numbers of target molecules.

## Reference genes other than *ABL1*

The published definitions of MR focus on the use of *ABL1* as a reference gene as this is used by the majority of laboratories worldwide.^15^ Of the principal alternative reference genes,^3^ *GUSB* is used by a significant minority of European laboratories, whereas *BCR* is used primarily in Australasia and some US laboratories. We have focused here on extending the MR definitions when *BCR-ABL1* is undetectable to include *GUSB*; further work will be required to extend these definitions to include *BCR*.

To determine the correspondence between *ABL1* and *GUSB*, we collected data from three centers that routinely analyzed the expression of both genes in parallel. We focused on CML samples that were <10% BCR-ABL^IS^ and had >10 000 *ABL1* copies. Of 1567 samples, the median ratio of *GUSB*/*ABL1* was 2.4 in the same volume of cDNA and therefore we consider that, for the purpose of defining deep MR, 10 000 *ABL1* transcripts are equivalent to 24 000 *GUSB* transcripts. The previously published^15^ definitions of MR can therefore be expanded as follows:

- MR^4^ (⩾4-log reduction from IRIS baseline)=either (i) detectable disease ⩽0.01% BCR-ABL^IS^ or (ii) undetectable disease in cDNA with 10 000–31 999 *ABL1* transcripts or 24 000–76 999 *GUSB* transcripts.
- MR^4.5^ (⩾4.5-log reduction from IRIS baseline)=either (i) detectable disease ⩽0.0032% BCR-ABL^IS^ or (ii) undetectable disease in cDNA with 32 000–99 999 *ABL1* transcripts or 77 000–239 999 *GUSB* transcripts.
- MR^5^ (⩾5-log reduction from IRIS baseline)=either (i) detectable disease ⩽0.001% BCR-ABL^IS^ or (ii) undetectable disease in cDNA with ⩾100 000 *ABL1* transcripts ⩾240 000 *GUSB* transcripts.

Although *GUSB* laboratories may use these definitions, we suggest that they should ideally derive their own correspondence between *ABL1* and *GUSB* (or other reference gene) using at least 50–100 remission (<10% BCR-ABL^IS^) samples to derive their own cutoffs for different MR levels. Before making this comparison, the amplification conditions should be optimized and in particular the amplification efficiency for the two genes should be the same. This can be achieved easily for *ABL1*, *GUSB* and *BCR* (and *BCR-ABL1*) using the ERM-AD623 plasmid.^17^ For laboratory-developed tests, we further recommend that ERM-AD623 is used directly as a qPCR calibrator for routine analysis or indirectly as a calibrator for in-house plasmid dilutions.

## Defining detectable and undetectable disease

There are several ways in which testing laboratories differ in how they define disease as detectable or undetectable. For individual amplification reactions and runs, we recommend that the established Europe Against Cancer criteria are used.^18^ In particular:

- The cutoff for positivity should correspond to a quantification cycle (Cq) of intercept +1 (which should generally lead to cutoffs of 41–42 Cq). In other words, samples with a Cq higher than intercept +1 should be considered as undetectable.
- The ‘no-template control' wells and reagent blanks should ideally not cross the threshold at any point but should certainly be at least 2 Cq above the intercept Cq for that run. If this is not the case, then the run must be considered as failed.

A major variable between centers is the number of replicate assays that are performed for each sample and the way that those replicates are considered to yield the final result. Typically, both *BCR-ABL1* and the reference gene are tested in duplicate, although some centers perform triplicate assays and some only perform single assays. If replicate assays are performed for *BCR-ABL1* (as recommended from RNA^19, 20^ or cDNA^21^ to help improve the accuracy of results) and any of the individual replicates are positive according to the criteria above, we recommend that the final result is considered as positive, that is, detectable disease. Even when testing in triplicate and two replicates are scored as undetectable and one is scored as detectable, the overall result should be scored as detectable or positive.

The Europe Against Cancer defines assay sensitivity by using normalized copy number and ΔΔCt methods, both of which relate the level of MRD to pretreatment levels for individual patients.^22^ This is not compatible with the IS in CML, which relates MRD levels to the IRIS standardized baseline, and therefore an alternative approach is required.

## Scoring MR when disease is detectable

In general, measurable residual disease^23^ should be assigned a value on the IS and scored as MR^4^ if ⩽0.01% BCR-ABL^IS^, MR^4.5^ if ⩽0.0032% BCR-ABL^IS^, and so on, provided that the sample fulfils the minimum quality criteria, that is, *ABL1* ⩾10 000 or *GUSB* ⩾24 000 in each replicate.^21^ If replicate analyses are performed and the values between replicates are comparable,^21^ then the number of *BCR-ABL1* and reference gene transcripts should be the total value across replicates and the final result expressed on the IS, that is, ((sum of *BCR-ABL1* copies)/(sum of reference gene copies)) × CF × 100 (see examples 1–3). As the reference gene in this context is used to estimate the amount of cDNA tested for *BCR-ABL1*, any difference in the number of replicates performed for *BCR-ABL1* and the reference gene will need to be taken into account (see example 4). In addition, we recommend that for scoring MR^4.5^, the total reference gene number should be 32 000–99 999 *ABL1* transcripts or 77 000–239 999 *GUSB* transcripts regardless of whether the disease is detectable or undetectable. For scoring MR^5^, the total reference gene number should be *ABL1* ⩾100 000 or *GUSB* ⩾240 000 (Table 1; see example 5).

Many centres score positive samples with a Cq higher than that of the lowest plasmid standard as ‘low-level positive', positive outside the quantifiable range, ‘<10 *BCR-ABL1*', if the lowest standard is 10, ‘<4 *BCR-ABL1*', if the lowest standard is 4, and so on. Indeed, some guidelines specifically recommend that values should not be estimated if they require extrapolation beyond the span of the standard plasmid calibration curve.^21^ This presents a problem for scoring low levels of disease and means, for example, that a laboratory using 10 as the lowest standard and a CF=1 would need to achieve *ABL1* reference gene values of 100 000 or greater to be able to score a sample with low-level detectable disease as MR^4^ and a value of ⩽320 000 to score a similar sample as MR^4.5^ (<10 *BCR-ABL1*/320 000 *ABL1*=⩽0.0032% *BCR-ABL1*). Despite the significant errors in quantifying small numbers of target molecules, we suggest that all low level-positive replicates should be assigned a specific number of *BCR-ABL1* transcripts by extrapolating below the lowest plasmid standard.

**Figure 1:**
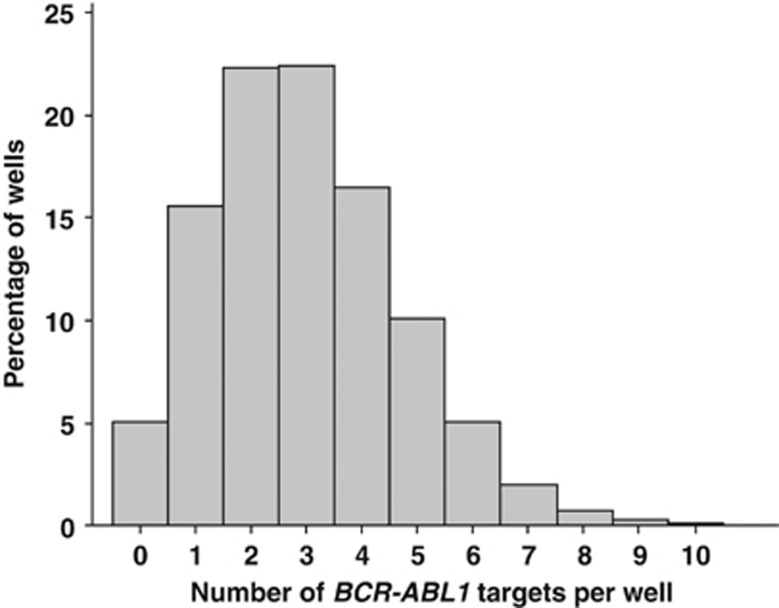
LoD of *BCR-ABL1* detection. The graph shows the Poisson distribution with a mean of 3 *BCR-ABL1* targets per well. The percentage of wells with 0–10 targets per well is indicated (20  000 computer-generated random datapoints; Minitab version 16, Coventry, UK) and shows that 95% of wells has at least one *BCR-ABL1* target. As the LoD is defined as the lowest concentration of target that can be detected with 95% confidence, the maximal theoretical LOD is 3 copies.

Testing laboratories have generally not rigorously determined their in-house limit of detection (LoD; defined as the lowest concentration of target that can be detected with 95% confidence) for *BCR-ABL1* transcripts. One reason for this is that standardized reagents have not been available to perform LoD analysis in a reproducible manner. Now, with the availability of the ERM-AD623 plasmid^17^ and other calibration reagents,^8^ we recommend that laboratories specifically measure^24^ and optimize their *BCR-ABL1* LoD. Clearly, the accuracy and precision by which MR can be scored critically depends on the *BCR-ABL1* LoD being maximized. A laboratory with a poor LoD may score a sample as undetectable and deep MR, whereas a laboratory with an optimized LoD may detect *BCR-ABL1* in the same sample and score it as not deep MR. Several studies have indicated that qPCR can be routinely optimized to detect single target molecules.^25, 26, 27^ Assuming that a single *BCR-ABL1* cDNA target can be detected and that there is no background signal (limit of blank=0), the LoD given by the Poisson distribution as three *BCR-ABL1* targets, as for a sample with an average of 3 targets/unit volume, there is a 95% chance that any unit volume will contain at least one target (Figure 1). Thus, we recommend that any replicate scored as positive should be assigned a value of ⩾3 *BCR-ABL1* copies, that is, positive replicates with estimated copy numbers of <3 should be scored as 3 (see examples 6–8). Alternative technologies, for example, digital PCR, are likely to be more accurate than qPCR for estimating small numbers of target molecules and may well become the method of choice for more accurate definition of low-level positive disease.^28, 29^

## Scoring MR when disease is undetectable

Analysis of multiple replicates can increase the sensitivity of detection simply by increasing the amount of sample that is tested. This approach has been used to design very sensitive assays to detect *BCR-ABL1* by qRT-PCR in healthy individuals,^30^ for genomic DNA-based tests for *BCR-ABL1* in CML,^31, 32, 33^ for detection of minimal residual disease in lymphoid disorders^25^ and for other applications such as noninvasive prenatal testing.^34^ When *BCR-ABL1* is undetectable in all replicates from the same sample, we recommend that the final result is given as (undetectable *BCR-ABL1*)/(sum of reference gene in all the replicates). We suggest that for routine analysis, a maximum of two or three replicates are performed (examples 9–11), although for specific studies it may be desirable to perform more replicates. Stringent quality criteria are essential, specifically replicates with <10 000 *ABL1* or <24 000 *GUSB* transcripts should be considered as inevaluable for determining deep MR (examples 12 and 13), and laboratories should maximize their LoD for *BCR-ABL1* to avoid false-negative results. As above, any difference in the number of replicates performed for *BCR-ABL1* and the reference gene should be taken into account (example 14).

## Examples

### *BCR-ABL1* detected in at least one replicate

Example 1 (Lab CF=0.8):

– *BCR-ABL1* replicate 1: detectable in 2 μl cDNA, estimated 7 copies.

– *BCR-ABL1* replicate 2: detectable in 2 μl cDNA, estimated 3 copies.

– *ABL1* replicate 1: 24 000 copies in 2 μl cDNA.

– *ABL1* replicate 2: 28 000 copies in 2 μl cDNA.

Result=(sum *BCR-ABL1*=10)/(sum *ABL1*=52 000) × 0.8 × 100=0.015%*=*MMR but not MR^4^.

Example 2 (Lab CF=1.8):

– *BCR-ABL1* replicate 1: undetectable in 5 μl cDNA.

– *BCR-ABL1* replicate 2: detectable in 5 μl cDNA, estimated 3 copies.

– *GUSB* replicate 1: 43 000 copies in 5 μl cDNA.

– *GUSB* replicate 2: 49 000 copies in 5 μl cDNA.

Result=(sum *BCR-ABL1*=3)/(sum *GUSB*=92 000) × 1.8 × 100=0.0059%*=*MR^4^.

*Comment*: Testing laboratories use different amounts of RNA to make cDNA, make different volumes of cDNA and use different volumes of cDNA for individual qPCR assays. The number of reference gene transcripts should be estimated in the same volume of cDNA used to test for *BCR-ABL1*. The use of other reference genes, for example, *BCR*, is possible, but the number of transcripts required to define different levels of MR remain to be determined.

Example 3 (Lab CF=0.5):

– *BCR-ABL1* replicate 1: undetectable in 5 μl cDNA.

– *BCR-ABL1* replicate 2: detectable in 5 μl cDNA, estimated 3 copies.

– *ABL1* replicate 1: 9000 copies in 5 μl cDNA.

– *ABL1* replicate 2: 8000 copies in 5 μl cDNA.

Result=inevaluable for MR.

*Comment*: Although the ((sum of *BCR-ABL1*)/(sum of reference gene)) × CF × 100 is <0.01%, the sample should be considered as inevaluable for the assessment of MR as the *ABL1* copy number in each replicate is <10 000.

Example 4 (Lab CF=0.8):

– *BCR-ABL1* replicate 1: undetectable in 5 μl cDNA.

– *BCR-ABL1* replicate 2: undetectable in 5 μl cDNA.

– *BCR-ABL1* replicate 3: detectable in 5 μl cDNA, estimated 4 copies.

– *ABL1* replicate 1: 14 000 copies in 5 μl cDNA.

– *ABL1* replicate 2: 15 000 copies in 5 μl cDNA.

Result=(sum *BCR-ABL1*=4)/(sum *ABL1*=29 000 × 1.5) × 0.8 × 100=0.0074%=MR^4^.

*Comment*: The sum of the reference gene copy number is multiplied by 1.5 (equivalent to multiplying the mean copy number × 3) because only two replicates were performed for the reference gene, whereas three replicates were performed for *BCR-ABL1*. In general, we consider that it is better to perform the same number of replicates for *BCR-ABL1* and the reference gene.

Example 5 (Lab CF=0.25):

– *BCR-ABL1* replicate 1: undetectable in 2 μl cDNA.

– *BCR-ABL1* replicate 2: detectable in 2 μl cDNA, estimated 3 copies.

– *ABL1* replicate 1: 12 000 copies in 2 μl cDNA.

– *ABL1* replicate 2: 14 000 copies in 2 μl cDNA.

Result=(sum *BCR-ABL1*=3)/(sum *ABL1*=26 000) × 0.25 × 100 =0.0029% sum of *ABL1* <32 000=MR^4^.

Comment: Although the ((sum of *BCR-ABL1*)/(sum of reference gene)) × CF × 100 is <0.0032%, the total *ABL1* value is <32 000 and should thus be considered as MR^4^. Considering the extreme examples of 31 999 *ABL1* transcripts and either 0 or 3 *BCR-ABL1* transcripts, it is apparent that this discrepancy only arises if the CF is <0.35 if using *ABL1* as a reference gene (or <0.82 if using *GUSB*).

Example 6 (Lab CF=0.8):

– *BCR-ABL1* replicate 1: undetectable in 5 μl cDNA.

– *BCR-ABL1* replicate 2: detectable in 5 μl cDNA, estimated 2 copies.

– *ABL1* replicate 1: 18 000 copies in 5 μl cDNA.

– *ABL1* replicate 2: 16 500 copies in 5 μl cDNA.

Result=(sum *BCR-ABL1*=3)/(sum *ABL1*=34 500) × 0.8 × 100=0.007%*=*MR^4^.

*Comment*: Each positive replicate should be assigned a value of ⩾3 copies and therefore the second *BCR-ABL1* replicate is scored as 3 copies.

Example 7 (Lab CF=0.8):

– *BCR-ABL1* replicate 1: detectable in 5 μl cDNA, estimated 2 copies.

– *BCR-ABL1* replicate 2: detectable in 5 μl cDNA, estimated 1 copy.

– *ABL1* replicate 1: 18 000 copies in 5 μl cDNA.

– *ABL1* replicate 2: 16 500 copies in 5 μl cDNA.

Result=(sum *BCR-ABL1*=6)/(sum *ABL1*=34 500) × 0.8 × 100=0.014%*=*MMR but not MR^4^.

*Comment*: Each positive replicate should be assigned a value of ⩾3 copies and therefore each *BCR-ABL1* replicate is scored as 3 copies.

Example 8 (Lab CF=0.8):

– *BCR-ABL1* replicate 1: detectable in 5 μl cDNA, estimated 2 copies.

– *BCR-ABL1* replicate 2: detectable in 5 μl cDNA, estimated 5 copies.

– *BCR-ABL1* replicate 3: detectable in 5 μl cDNA, estimated 7 copies.

– *ABL1* replicate 1: 34 000 copies in 5 μl cDNA.

– *ABL1* replicate 2: 38 500 copies in 5 μl cDNA.

– *ABL1* replicate 3: 32 500 copies in 5 μl cDNA.

Result=(sum *BCR-ABL1*=15)/(sum *ABL1*=105 000) × 0.8 × 100**=**0.011%*=*MMR but not MR^4^.

*Comment*: Each positive replicate should be assigned a value of ⩾3 copies.

### *BCR-ABL1* undetected in all replicates

Example 9:

– *BCR-ABL1* replicate 1: undetectable in 5 μl cDNA.

– *BCR-ABL1* replicate 2: undetectable in 5 μl cDNA.

– *ABL1* replicate 1: 16 500 copies in 5 μl cDNA.

– *ABL1* replicate 2: 18 000 copies in 5 μl cDNA.

Result=undetectable *BCR-ABL1* in 34 500 *ABL1*=MR^4.5^.

Example 10:

– *BCR-ABL1* single analysis: undetectable in 5 μl cDNA.

– *ABL1* single analysis: 45 000 copies in 5 μl cDNA.

Result=undetectable *BCR-ABL1* in 45 000 *ABL1*=MR^4.5^.

*Comment*: Although single analyses are performed by some centers, replicate assays from RNA or cDNA improves the accuracy of results.^19, 20, 21^

Example 11:

– *BCR-ABL1* replicate 1: undetectable in 2 μl cDNA.

– *BCR-ABL1* replicate 2: undetectable in 2 μl cDNA.

– *BCR-ABL1* replicate 3: undetectable in 2 μl cDNA.

– *ABL1* replicate 1: 24 000 copies in 2 μl cDNA.

– *ABL1* replicate 2: 22 500 copies in 2 μl cDNA.

– *ABL1* replicate 3: 24 000 copies in 2 μl cDNA.

Result=undetectable *BCR-ABL1* in 70 500 *ABL1*=MR^4.5^.

Example 12:

– *BCR-ABL1* replicate 1: undetectable in 5 μl .

– *BCR-ABL1* replicate 2: undetectable in 5 μl cDNA.

– *ABL1* replicate 1: 7000 copies in 5 μl cDNA.

– *ABL1* replicate 2: 8000 copies in 5 μl cDNA.

Result=inevaluable for MR as *ABL1* <10 000 in each replicate.

Example 13:

– *BCR-ABL1* replicate 1: undetectable in 5 μl cDNA.

– *BCR-ABL1* replicate 2: undetectable in 5 μl cDNA.

– *ABL1* replicate 1: 6000 copies in 5 μl cDNA.

– *ABL1* replicate 2: 14 000 copies in 5 μl cDNA.

Result=inevaluable for MR.

*Comment*: One replicate is <10 000 *ABL1* and hence the sample should be considered as inevaluable for MR. As the two *ABL1* replicates are discordant, the reference gene qPCR could be repeated.

Example 14:

– *BCR-ABL1* replicate 1: undetectable in 2 μl cDNA.

– *BCR-ABL1* replicate 2: undetectable in 2 μl cDNA.

– *BCR-ABL1* replicate 3: undetectable in 2 μl cDNA.

– *ABL1* replicate 1: 16 500 copies in 2 μl cDNA.

– *ABL1* replicate 2: 18 000 copies in 2 μl cDNA.

Result=undetectable *BCR-ABL1* in (34 500 × 1.5=51 750 *ABL1*)=MR^4.5^.

*Comment*: The sum of the reference gene copy number is multiplied by 1.5 because only two replicates were performed for the reference gene, whereas three replicates were performed for *BCR-ABL1*.

## Concluding remarks

The remarkable progress in the treatment of CML has demanded definitions of deep MR that are stretching the technology of molecular monitoring to its limit. The recommendations described here are an attempt to develop standardized laboratory approaches that strike a reasonable balance between scientific accuracy and clinical reality. It should be recognized that there is considerable inherent uncertainty in defining very low levels of disease and that it will be important to continue to look at trends over time to recognize sustained MR. Furthermore, the reproducible application of the recommendations depends critically on the ability of testing laboratories to measure absolute numbers of reference gene transcripts in a comparable manner, as well as their ability to maximize the LoD for *BCR-ABL1* and minimize interassay variability. It is obvious that future methodological improvements that increase the amount of sample tested (as determined by the number of reference gene transcripts) will increase the precision and accuracy of scoring MR^4^ or MR,^4.5^ as well as enabling even deeper levels of MR to be determined.

We recognize that these recommendations may need to be adapted to local requirements and changing technologies. We also recognize that laboratory recommendations in isolation are meaningless and that the critical question is the clinical significance of achieving deep levels of MR. We anticipate that the standardized definitions described here will help to progress clinical studies that aim ultimately to cure CML as well as providing a common framework for reporting routine results.

## Figures and Tables

**Table 1:** Summary of reference gene numbers required for scoring deep molecular response

	*MR*^*4*^	*MR*^*4.5*^	*MR*^*5*^
Minimum sum of reference gene transcripts irrespective of whether *BCR-ABL1* is detected or not^a^	10 000 *ABL1* 24 000 *GUSB*	32 000 *ABL1* 77 000 *GUSB*	100 000 *ABL1* 240 000 *GUSB*
BCR-ABL^IS^ level for positive samples^b^	⩽0.01%	⩽0.0032%	⩽0.001%

a Numbers of reference gene transcripts in same volume of cDNA that is tested for *BCR-ABL1*. The minimum number in any individual replicate should be 10 000 *ABL1* or 24 000 *GUSB*.

b Provided that the minimum reference gene copy numbers in the row above are fulfilled.
